# Pirin1 (PRN1) Is a Multifunctional Protein that Regulates Quercetin, and Impacts Specific Light and UV Responses in the Seed-to-Seedling Transition of *Arabidopsis thaliana*

**DOI:** 10.1371/journal.pone.0093371

**Published:** 2014-04-04

**Authors:** Danielle A. Orozco-Nunnelly, DurreShahwar Muhammad, Raquel Mezzich, Bao-Shiang Lee, Lasanthi Jayathilaka, Lon S. Kaufman, Katherine M. Warpeha

**Affiliations:** 1 Molecular, Cell and Developmental Group, Department of Biological Sciences, Department of Biological Sciences, University of Illinois at Chicago (UIC), Chicago, Illinois, United States of America; 2 Protein Research Laboratory, University of Illinois at Chicago (UIC), Chicago, Illinois, United States of America; Wake Forest University, United States of America

## Abstract

Pirins are cupin-fold proteins, implicated in apoptosis and cellular stress in eukaryotic organisms. Pirin1 (PRN1) plays a role in seed germination and transcription of a light- and ABA-regulated gene under specific conditions in the model plant system *Arabidopsis thaliana*. Herein, we describe that PRN1 possesses previously unreported functions that can profoundly affect early growth, development, and stress responses. *In vitro*-translated PRN1 possesses quercetinase activity. When PRN1 was incubated with G-protein-α subunit (GPA1) in the inactive conformation (GDP-bound), quercetinase activity was observed. Quercetinase activity was not observed when PRN1 was incubated with GPA1 in the active form (GTP-bound). Dark-grown *prn1* mutant seedlings produced more quercetin after UV (317 nm) induction, compared to levels observed in wild type (WT) seedlings. *prn1* mutant seedlings survived a dose of high-energy UV (254 nm) radiation that killed WT seedlings. *prn1* mutant seedlings grown for 3 days in continuous white light display disoriented hypocotyl growth compared to WT, but hypocotyls of dark-grown *prn1* seedlings appeared like WT. *prn1* mutant seedlings transformed with GFP constructs containing the native *PRN1* promoter and full ORF (*PRN1*::PRN1-GFP) were restored to WT responses, in that they did not survive UV (254 nm), and there was no significant hypocotyl disorientation in response to white light. *prn1* mutants transformed with *PRN1*::PRN1-GFP were observed by confocal microscopy, where expression in the cotyledon epidermis was largely localized to the nucleus, adjacent to the nucleus, and diffuse and punctate expression occurred within some cells. WT seedlings transformed with the *35S*::PRN1-GFP construct exhibited widespread expression in the epidermis of the cotyledon, also with localization in the nucleus. PRN1 may play a critical role in cellular quercetin levels and influence light- or hormonal-directed early development.

## Introduction

Flavonoids are a class of phenylpropanoids, that are induced in germinating seedlings by UV and blue-light (B) [1], and are reported to play many diverse, but not fully understood, roles in plant physiology [2], [3]. Quercetin (a flavonol, a sub-class of the flavonoids) accumulates in young Arabidopsis seedlings of 4–7 days (d) old, particularly in the cotyledonary node, hypocotyl/root transition zone and root tip, with glycosylated flavonoids in the cotyledon [4]. *In vivo* evidence indicates that flavonols regulate auxin accumulation in the *transparent testa 4* (*tt4;* makes no flavonols, including kaempferol and quercetin) mutants [5], [6]. It has also been reported that auxin accumulates in *rol1-2* (*repressor of lrx1*) mutant seedlings due to flavonol-induced changes to auxin transport [7]. Comparing auxin transport in *tt4* and *tt7* (makes kaempferol but not quercetin) mutants, it was shown that derivatives of quercetin can inhibit basipetal auxin transport, elongation, and gravitropism [8]. Quercetin due to its distinct structure can function as an antioxidant and UV screening compound [3], [9]. The specific functions of flavonols in the seed-to-seedling developmental transition are still poorly understood, and may involve protein(s) that are still undescribed. Quercetin may also play important roles within the plant cell. Saslowsky et al. demonstrated that flavonoids and specific biosynthetic enzymes of flavonoids were present both in the nucleus and cytosol [10], and Peer et al., found quercetin in the nuclear region, endomembrane system and plasma membrane [4].

Quercetin is cleaved by quercetinase proteins, resulting in carbon monoxide and 2-protocatechuoylphloroglucinol carboxylic acid [11]. Adams and Jia demonstrated that the protein Pirin (PRN or PIR) of both human and bacteria have quercetinase activity *in vitro* [12]. Pirins have rapidly become a focus of interest given reported roles in metabolism [13], apoptosis [14], [15], cellular stress [16] and malignancy [17], [18]. Pirins are highly conserved members of the cupin super-family [19] found in prokaryotes, fungi, plants, and expressed at low levels in all examined cell types in mammals [20]. Pirin was originally identified as a transcription co-factor, interacting with the heterotrimeric nuclear factor I/CCAAT box transcription factor NFY (*aka* NFI/CTF1; HAP) to drive adenovirus DNA replication and polymerase II transcription [20].

In plants, pirin investigations have been limited to specific periods of the life cycle (i.e. germination, and transition from vegetative to reproductive growth) [21]. Pirin was first identified in plants by Orzaez et al. in tomato, as potentially being involved in programmed cell death [15]. Arabidopsis Pirin1 (PRN1) was found via yeast-2-hybrid screening using the G-protein-α subunit (GPA1) as bait, and was shown to have a specific role in ABA regulation of germination in *Arabidopsis thaliana* [21]. PRN1 has been reported to play roles in several specific contexts, such as the B-induction of *LhcB* expression [22] and defense against seedling infection by *Cryptococcus* fungi as inferred by T-DNA insertion mutant analysis [23]. *PRN1* is also reported in high-throughput data derived from carbon status changes [24], meta-analysis of microarrays of plant hormone regulation [25], and in expression analysis where *PRN1* is induced by drought [26]. Although PRN1 is implicated in aspects of abiotic and biotic physiology, its different potential activities of transcription cofactor and possible quercetinase activity have yet to be reconciled.

G proteins are well known to be involved in a number of plant responses to various stimuli [27]. To date in Arabidopsis, GPA1 and PRN1 are reported in regulating germination [21]; and GPA1, PRN1 and NFY form a signal transduction chain, responsible for B-transcription of *LhcB* in 6-d-old dark-grown (etiolated) seedlings [22]. In earlier studies on PRN1, we had focused on a simple developmental period, from 0 (seed) to 6-7-d-old, utilizing only completely etiolated Arabidopsis seedlings [22]. However, study of *prn1* mutants and infection with fungi in dim and bright sunlight conditions has indicated that PRN1 may play a role in photomorphogenic transition and development, as well as in defense [23].

Given PRN1’s reported effects on seed-to-seedling development [21], [22], and its potential action in stress/defense [23], the activities of this protein are still largely unknown. Based on what is reported for human and bacterial pirins, we hypothesized that PRN1 may have a similar capability to cleave quercetin. Secondly, we hypothesized if PRN1 can regulate quercetin levels, it may affect growth of the young seedling, considering past reports of quercetin’s influence on auxin accumulation and transport. To explore PRN1’s specific effects, we conducted quercetinase enzyme assays with *in vitro* translated PRN1, we observed phenotypes in seedling development (in light and darkness) and stress responses by using a T-DNA insertion mutant of *PRN1*, and explored native promoter *PRN1-* and *35S-*driven expression of PRN1 in transgenic seedlings. We explored responses of seedlings grown in complete darkness, and under white light growth conditions (continuous white light for 3 d; and 16∶8 light:dark for 6 d). We focused on the cotyledon and hypocotyl due to the most prominent phenotypes observed. The data considered support the hypotheses that PRN1 may regulate quercetin levels in at least the epidermal layer cells of the cotyledon, and PRN1 may play a role in light regulation of hypocotyl growth and orientation.

## Materials and Methods

### Chemicals

All chemicals, unless otherwise noted, were obtained from Sigma (St. Louis, MO).

### Plant Materials, Seed Stocks and Accessions

Seeds of wild type (WT) Columbia *Arabidopsis thaliana* and mutants carrying a T-DNA insertion within the coding region of *PRN1* (SALK_006939), and the coding region of *ADT3* (published also as *PREPHENATE DEHYDRATASE* [*PD1*]), but referred to as *AROGENATE DEHYDRATASE* [*ADT3*] after description of the ADT family, henceforth is referred to as *ADT3*; SALK_029949) were obtained from the Arabidopsis Biological Resource Center (Columbus, OH) [28]. The mutant lines are homozygous null for the reported insertions. Plants intended for seed stocks were grown in Scott Metromix 200 (Scotts; Marysville, OH) in continuous white light [21]. Sequence data from this article can be found in the EMBL/GeneBank data libraries under accession numbers At2g27820 (ADT3), At3g59220 (PRN1).

### Plant Growth Conditions for Experiments

Seedlings of *Arabidopsis thaliana* WT or T-DNA insertion mutants were grown on 0.5× Murashige and Skoog medium, 0.8% agarose phytatrays as described [21]. The growth medium contained no added sugars, hormones, vitamins or other nutrients. For all experiments, seedlings were sterilized in a bleach solution, all sterilization, planting and manipulations were carried out under dim green light [29]. Seeds were then washed in sterile water, mixed with low melt agarose then sown on phytatrays, and subsequently sealed in light-proof black plastic boxes. Planted seeds were stratified for 48 h in complete darkness at 4°C as described [29] without a light treatment. Cold-vernalized seeds were then moved to appropriate dark (dark-grown = continuous darkness) and/or light conditions (phytatrays or vertical trays moved from dark boxes to open boxes), detailed below. Seedlings were grown between 3 d and 7 d for most experiments in either complete darkness or in white light (continuous or 16∶8, 10^2^ μmol m^−2^ s^−1^) chambers as described, at 20°C. Vertical growth experiments were as described here. Seeds were prepared as described for phytatrays, except no top agarose was used, with sterilized and washed seeds set directly onto the 0.5× medium (same as phytatray medium). Plates were sealed with parafilm then taped in vertical position in black plastic boxes, were stratified for 48 h in complete darkness at 4°C, then moved to the appropriate growth condition for the experiment.

### Determination of Quercetin Levels

6-d-old dark-grown seedlings were given a 10^4^ μmol m^−2^ total dose of 317 nm UV, as quercetins are known to be induced by UV-B [9]. Six h later the aerial portions of sets of seedlings (1 full phytatray of seedlings per sample; 100 μL of dry seed sown/phytatray) were harvested directly into liquid nitrogen under dim green light, then finely ground, with similar sample preparation as described [29], except the final dried samples were weighed, then further purified by dissolving them in water and extracting with ethyl acetate. The ethyl acetate layer was dried and re-dissolved in a water/methanol mixture for analysis. Liquid chromatography-mass spectrometry analysis of hydrolyzed quercetin and kaempferol was carried out using an Agilent 6410 Triple Quad mass spectrometer (Agilent Technologies; Santa Clara, CA) coupled with an Agilent HPLC 1200 series chromatographic system. The system was operated using Agilent Mass-Hunter workstation software. Separation was achieved on a column HPLC chip (Agilent G4240, C18, 300A°, 43 mm chip) using water and methanol as the mobile phase. The mobile phase was pumped at a 500 nL/min flow rate. The initial mobile phase consisted of 50% methanol for 1 min, and the amount of methanol was increased linearly to 90% over 2.5 min. A multiple-reaction motoring method in the negative ion mode was used for the analysis of quercetin (m/z 301.1−>121.1) and kaempferol (m/z 285.1−>117.1). Values were determined as ng/g liquid nitrogen ground weight. Resultant data obtained from each data pair (WT vs. *prn1*) were analyzed by a two-tailed T test, where data was entered into GraphPad to determine SD of the ratios for 4 replicates.

### Synthesis of PRN1 and GPA1 Protein

Full-length PRN1 and GPA1 templates were prepared, amplified and purified as described previously [21]. PRN1 and GPA1 protein were separately produced in coupled *in vitro* transcription/translation reactions using the TNT T7 Coupled Wheat Germ Extract System (Promega; Madison, WI) as directed, with a 90 min translation time at 30°C, and as previously described, with 50% concentration of extract by using a microcentrifugation concentrating filter to maximize retention of protein 30 kD and above (Millipore, Billerica, MA) [29].

### Quercetinase Assay

For a final volume of 500 μL, freshly *in vitro*-translated PRN1 extract (16 μg) was incubated with a final concentration of 10 μM quercetin (diluted from 5.0 mM DMSO stock immediately before assay) in a total reaction volume including 1 μL of 0.5× MS medium, and utilizing the buffer and method of quercetinase determination of activity described in the assay of Adams and Jia [12]. For G-protein assays, 1 μM MgCl_2_ and 150 mM NaCl were additionally included in the assay buffer. Reactions were carried out in darkness, in a 26°C circulating water bath for 15 min; then the quercetin 2,3-dioxygenase activity of PRN1 was determined by measuring absorbance as described [12], using a Perkin Elmer scanning spectrophotometer (Perkin Elmer; Waltham, MA).

### In vitro GPA1-PRN1 Activity Assay

GPA1 was locked in either the active or inactive confirmation by using non-hydrolysable analogs, in a pre-incubation of 16 μg of the *in vitro* translation GPA1 product extract with a final concentration of 100 μM GTPγS (non-hydrolysable GTP analog), or GDPβS (non-hydrolysable GDP analog) in HEPES, pH 7.5, in 100 μL total volume, overnight in darkness at 10°C under rotation [29]. The “activated” and “inactivated” GPA1 mixtures were then separately incubated with PRN1 in a 1∶1 volume ratio (26°C for 15 min, circulating water bath) and then tested for quercetinase activity as described above. The ‘buffer only’ scan was subtracted from all spectra.

### UV-C Kill Assay

Seeds were planted and seedlings grown as described above. After the cold-stratification, the seedlings were transferred to complete darkness. UV-C treatments were administered on d 6 as previously described [30] with 4 or 8 min of overhead (254 nm) radiation administered. Seedlings were returned to complete darkness for 24 h. UV-C killing doses for *adt3* or WT were determined by dose-response ‘titration’ experimentation. Seedlings were photographed from the side.

### Light-grown Hypocotyl Orientation Assay

Seeds were planted and grown on phytatrays as described above. After cold-stratification, the seedlings were transferred to continuous white light. After 72 h, they were photographed from the side using a dissection microscope, and hypocotyl angle (in reference to the horizontal plane) was measured. Two sets of 30–35 seedlings were planted, then, due to highly similar means at time of observations, data were pooled for analysis. For the complementation experiments, the seedlings grown on phytatrays were photographed from overhead at 72 h after stratification. Different sets of seeds were planted and grown on vertical plates in both darkness and 16∶8 light conditions for 6 d, then were photographed to observe the seedling phenotype(s). Dissection microscope images from vertical plate seedlings were reassembled using a stitching plugin for ImageJ [31].

### Cloning

Standard molecular biology techniques and the Gateway system (Invitrogen) were used for all cloning procedures. PCR fragments were created using the primers, shown in Table S1. WT Arabidopsis genomic DNA was used to generate the *PRN1* promoter fragment, and WT Arabidopsis cDNA was used to generate the PRN1 ORF fragment. The *PRN1* full promoter fragment was then cloned into the Invitrogen pDONR P4-P1R vector, and the *PRN1* ORF fragment was cloned into the Invitrogen pENTR/D-TOPO vector. LR reactions (Invitrogen) were then performed with verified entry clones to obtain expression clones. The pGreen [32] binary vector derivative containing a NOS terminator with a C-terminal GFP fusion and spectinomycin and BASTA resistance genes [33] was used for the native promoter construct (*PRN1*::PRN1-GFP). The pEarleyGate 103 [34] vector containing an OCS terminator with a *35S* promoter, a C-terminal GFP-His fusion and kanamycin and BASTA resistance genes was used for the overexpression construct (*35S*::PRN1-GFP). All constructs were confirmed via restriction enzyme digest, PCR, and sequencing. Verified expression clones were transformed in *prn1* mutant or WT backgrounds via floral dip [35]. Transformed seeds/seedlings were grown under BASTA selection. Homozygous transformed seedlings were selected in the third generation (T3) for the native promoter construct (*PRN1*::PRN1-GFP) in *prn1* background, and at T2 for the overexpression construct (*35S*::PRN1-GFP) in WT background.

### Visualization of Transgene Expression

Transgenic and untransformed 6-d-old dark-grown seedlings were fixed using paraformaldyhyde (2.5%), then were mounted in Prolong Gold antifade reagent with DAPI (Life Technologies: Grand Island, NY) on glass slides. Seedlings were harvested in dim green light into fixative, then all other manipulations were done in lab lighting. Images were obtained using an Andor WD Spinning Disk confocal system (Yokagawa CSU-W1 with standard 50 μm pinholes) using iQ2 software. For each replicate 12–20 whole seedlings were screened and areas of fluorescence examined in 1.0 μm thick horizontal sections, where z slices were collected through the complete seedling. Exposures were identical among samples, and contrast uniform on each channel using 3 solid-state lasers. All wavelengths were detected sequentially using narrow band pass filters, basically eliminating any potential for crosstalk. DAPI was stimulated using a 405 nm diode laser (200 ms exposure, laser intensity 64%), and detected with Semrock Brightline, Single Band Fluorescence Filter, 447 Centre; GFP was stimulated with a 488 nm diode laser (400 ms exposure, laser intensity 48.4%), and detected with Semrock Brightline, Single Band Fluorescence Filter, 525 Centre; red fluorescing molecules (largely protochloryllides so etioplasts were identifiable in samples) were stimulated with a 561 nm diode laser (350 ms exposure, laser intensity 67.7%), and detected with Semrock Brightline, Single Band Fluorescence Filter, 607 Centre. At least three separate transgenic lines were used to confirm all reported expression data. Images were prepared with ImageJ software (NIH, Bethesda, MD).

### Epidermal Peel

Cotyledon epidermal peels were conducted as described [36], with specific modifications. Hollister 7730 medical adhesive (Hollister, Libertyville, IL) was sprayed across a glass slide, then waved in air to remove bubbles. After 5 min, live 6-day seedling cotyledons were set adaxial side down, then pressed into adhesive by parafilm to ensure evenly adhered surface. Cells were scraped away with a curved microspatula 3 min later. Prolong Gold antifade reagent with DAPI stain was set on top of the cells for 5 min, then cover slides were mounted for analysis via spinning disk confocal microscopy. *prn1* mutants or *prn1*-transformed with *PRN1*::PRN1-GFP or WT-transformed with *35S*::PRN1-GFP were viewed on spinning disk confocal after DAPI-stain was applied. 10–15 cotyledons were viewed per replicate of 3 independent replicates, utilizing the same lasers and imaging conditions described above for visualization of transgene expression.

### Statistics

Data shown in figures were entered into Prism v. 5.0 (GraphPad Software, Inc., GraphPad.com), where mean and SD or SEM are shown for most graphic depictions. For enzymatic activity curves SD is shown on the figures. For bar graphs, quercetin quantification (ng/g liq nitrogen ground mass) is shown as ng/g. The WT and *prn1* samples where planted, harvested and extracted as pairs where they were compared, respectively. A two-tailed T test was used with significance set at p<.05. SD are shown on the relevant Figure. For hypocotyl orientation data, a non-parametric statistical analysis was performed for all data. The Kolmogorov-Smirnov normality test indicated that the WT and *prn1* data were non-normally distributed. Subsequently, for the angle of hypocotyl orientation, a Mann-Whitney test was used to compare the *prn1* hypocotyl angle to the WT angle, where on the figure SEM is shown.

## Results

### PRN1 Possesses Quercetinase Activity

**Figure 1:**
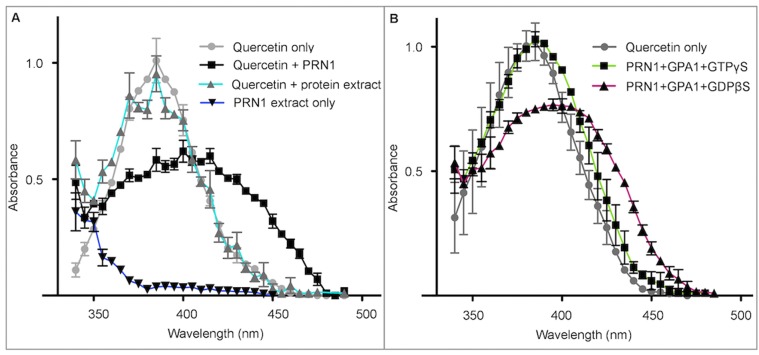
*In vitro*-translated PRN1 has quercetinase activity. **A. *in-vitro*-translated PRN1 possesses quercetinase activity.** *In vitro*-translated PRN1 was incubated with a final concentration of 10 μM quercetin in a 500 μL total reaction volume, or as a control, 10 μM quercetin alone, in buffer as described [12]. Reactions were carried out in darkness in a 26°C water bath for 15 min. After incubation at 26°C, absorption spectra of quercetin alone (Quercetin only), Quercetin+PRN1, Quercetin+protein extract, or PRN1 extract only were determined using a Perkin Elmer scanning spectrophotometer (Perkin Elmer; Waltham, MA). n = 3, SD shown. **B. The conformational status of GPA1 affects the quercetinase ability of PRN1.** *In vitro*-translated GPA1 was pre-incubated with non-hydrolyzable analogs (either GTPγS or GDPβS) overnight at 10°C, then was incubated with PRN1, then (PRN1+GPA1+GTPγS or PRN1+GPA1+GDPβS) was added to buffer+quercetin to assess the effects on quercetinase activity as shown. Absorption spectra of quercetin alone (Quercetin only) also shown. n = 3, SD shown.

Based on reports that *in vitro*-translated human and bacterial Pirin could act as quercetinases [12], *in vitro*-translated PRN1 was analyzed for the ability to cleave quercetin (Figure 1A). Utilizing the buffer of Adams and Jia [12], quercetin has a reported absorbance maximum of 384 nm, which we observed herein for quercetin (Figure 1A). Upon incubation with *in vitro*-translated PRN1, the absorbance maximum shifted to ∼405–410 nm by 15 min at 26°C, indicating cleavage of quercetin [12], and confirming that nascent PRN1 could potentially function as a quercetinase (Figure 1A). Incubation of quercetin with the *in vitro* translation components alone did not result in absorbance changes (Figure 1A).

### Quercetinase Activity is Observed When GPA1 is in the Inactive Conformation

To address potential regulation of the quercetinase activity of the nascent PRN1 protein obtained by *in vitro* translation, we explored the interactions between GPA1 and PRN1 (original interaction reported by [21], further explored by [22], and observed in high-throughput yeast-2-hybrid assays [37]). We pre-incubated *in vitro*-translated GPA1 with GTPγS (non-hydrolyzable GTP analog) or GDPβS (non-hydrolyzable GDP analog), in order to produce GPA1 maintained in an activated (GTPγS) or inactivated (GDPβS) conformation, respectively. GPA1 in the active or inactive confirmation was then incubated with *in vitro*-translated PRN1 and quercetin. The resulting absorbance profiles (Figure 1B) indicated that GPA1 in its inactive confirmation permits quercetinase activity of PRN1. When GPA1 was maintained in its active confirmation no quercetinase activity was measurable.

### *prn1* Mutants can Produce Stable Elevated Amounts of Quercetin Induced by Brief UV-B Treatment

**Figure 2:**
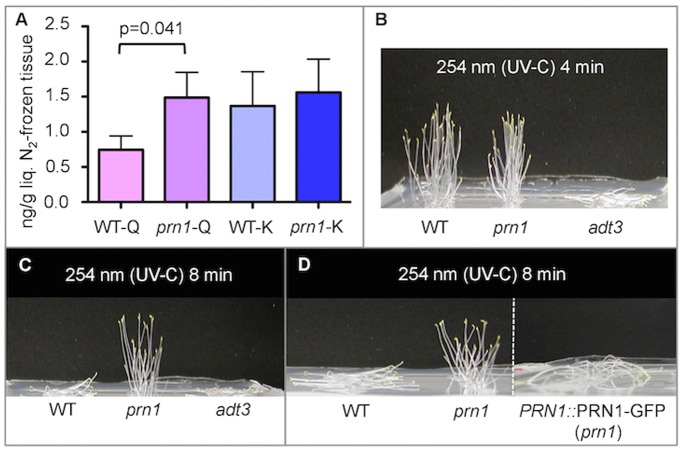
*prn1* mutants accumulate excess quercetin, and can survive UV-C irradiation that kills WT. **A. *prn1* mutants accumulate more quercetin compared to WT seedlings.** 6-d-old dark-grown seedlings were treated with a brief pulse of UV (317 nm), returned to darkness, then harvested 6 h later to determine the total extractable quercetin (Q) or kaempferol (K) in WT or *prn1* seedlings (WT-Q; *prn1*-Q; WT-K; *prn1*-K). The levels of Q and K are indicated for *prn1* and WT and SD are shown. n = 4 **B & C.** ***prn1*** **mutants survive a UV-C radiation treatment that kills WT seedlings.** Seedlings grown in complete darkness as described in methods. UV-C treatments were administered on d 6 as described [30], with 4 min (B) or 8 min (C) of 254 nm (UV-C) radiation. Seedlings were returned to complete darkness for 24 h. UV-C killing doses for *adt3* or WT were determined by prior ‘titration’ experimentation [30]. Seedlings were photographed from the side. n = 4. **D.** ***prn1*** **seedlings transformed with** ***PRN1*** **::PRN1-GFP construct are killed by 8 min of UV-C, similar to WT.** Seeds (30) of WT, *prn1* or *prn1* transformed with *PRN1*::PRN1-GFP (*PRN1*::PRN1-GFP *prn1*) were sown, then seedlings grown in complete darkness. UV-C treatments were administered on d 6 as described [30], with an 8 min dose of 254 nm (UV-C) radiation. Seedlings were returned to complete darkness for 24 h. Seedlings were photographed from the side. n = 4.

To further explore the possible role of PRN1 as a quercetinase in plants, etiolated, 6-d-old Arabidopsis WT and *prn1* seedlings were both irradiated by UV-B (317 nm), then 6 h post-irradiation, aerial portions of seedlings were harvested. Levels of extractable hydrolyzed quercetin, as well as the closely related compound kaempferol were quantified (Figure 2A). Quercetin levels in *prn1* mutants were approximately twice as high as those detected in WT, while kaempferol levels remained effectively unchanged (Figure 2A). This finding supports the results of the *in vitro* quercetinase assays, suggesting that if PRN1 can act as a quercetinase, lack of PRN1 may result in elevated quercetins in mutant seedlings, since there may be no mechanism for cleavage of quercetin to maintain a WT level.

### *prn1* Mutants can Survive UV-C Radiation That Kills WT

If young, dark-grown *prn1* seedlings potentially have more or accumulate more quercetin, then one would expect the *prn1* mutants to exhibit improved resistance to cellular stress caused by high energy UV (UV-C; 254 nm). We confirmed this hypothesis by using a published assay [30], where we exposed WT and *prn1* insertion mutants to a brief dose of UV-C, then returned the seedlings to darkness (Figures 2B & 2C). 24 h after the UV-C treatment, WT seedlings survived, while radiation-sensitive *adt3* (T-DNA insertion of AROGENATE DEHYDRATASE 3, sensitive to radiation as prior reported [30]) seedlings died, and *prn1* mutant seedlings survived (Figure 2B). When the UV-C dose was doubled, WT seedlings died, and *prn1* seedlings not only survived, but also exhibited increased hypocotyl shortening (Figure 2C compared to 2B). *prn1* seedlings (T3, homozygous) transformed with a native *PRN1* promoter and PRN1 ORF construct were killed by the 8 min of UV-C, complementing the *prn1* mutation (Figure 2D).

### *prn1* Mutants Exhibit an Altered Angle of Hypocotyl Growth When Grown in White Light

**Figure 3:**
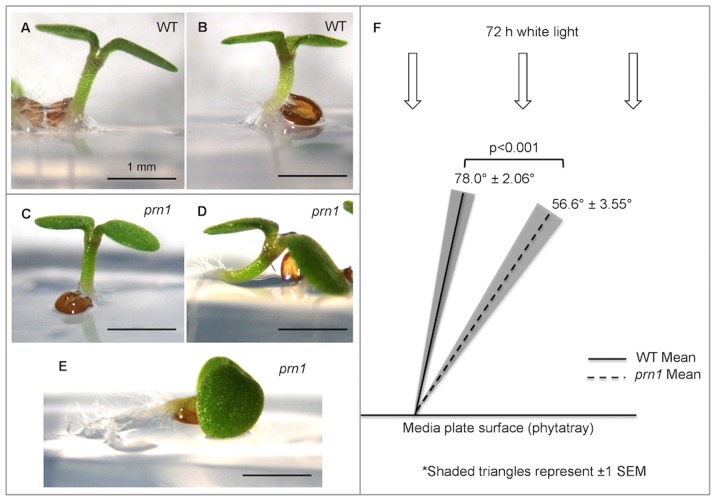
3-d-old light-grown *prn1* mutants exhibit abnormal light-grown shoot orientation phenotype. Three (72 h)-d-old white light-grown seedlings were photographed from the side using a dissecting microscope to compare WT (**A–B**) and *prn1* mutant (**C–E**) seedlings. Images are representative of the range of orientations observed. Scale bars each represent 1.0 mm. Hypocotyl angles were measured for individual seedlings in reference to the horizontal phytatray surface (where vertical = 90°) and mean angles were calculated (**F**). Means are shown by lines (solid for WT; dotted for *prn1*) and SEMs are represented by shaded triangles. n = 65. SEM shown.

Since flavonoids are induced in seedlings by light [1], we also observed the growth of *prn1* and WT seedlings grown under continuous white light for 3 d on phytatrays. *prn1* mutants exhibited an average hypocotyl growth angle (from the horizontal) of 56.6 (+/−3.55 SEM) degrees, while the WT seedlings exhibited an average of 78.0 (+/−2.06 SEM) degrees (Figure 3). Examples of the phenotype are shown (Figure 3A–E). Since the Kolmogorov-Smirnov normality test indicated that the WT and *prn1* data were not normally distributed (WT: KS = 0.222 and p<0.010, *prn1*: KS = 0.146 and p<0.010; Figure S1A and S1B, respectively), a non-parametric statistical analysis was performed. We found that the *prn1* shoot growth angle was significantly different than the WT angle (Mann-Whitney test; p<0.001) (Figure 3F). The disoriented hypocotyl phenotype was complemented (oriented like WT) in *prn1* seedlings transformed by the native construct (*PRN1*::PRN1-GFP; Figure S1C). No gross phenotypic differences were observed for *prn1* seedlings compared to WT seedlings from 6-d-old dark-grown conditions on vertical plates (Figure S2). For 6-d-old light-grown (16∶8) seedlings on vertical plates (Figure S3), we observed a hypocotyl phenotype similar to that reported for 3-d continuous white light *prn1* seedlings (Figure 3), and also observed shorter roots, which was complemented by transformation with the native promoter construct (*PRN1*::PRN1-GFP) (Figure S3). However, when WT were transformed with *35S*::PRN1-GFP, the hypocotyl phenotype was reminiscent of the deficiency (*prn1* mutant seedlings), but with noticeably smaller cotyledons, hypocotyl and root (Figure S3).

### PRN1-GFP Fluorescence is Observed in Cells of the Epidermis, Associated with the Nucleus in 6-d-old Dark-grown Seedlings

**Figure 4:**
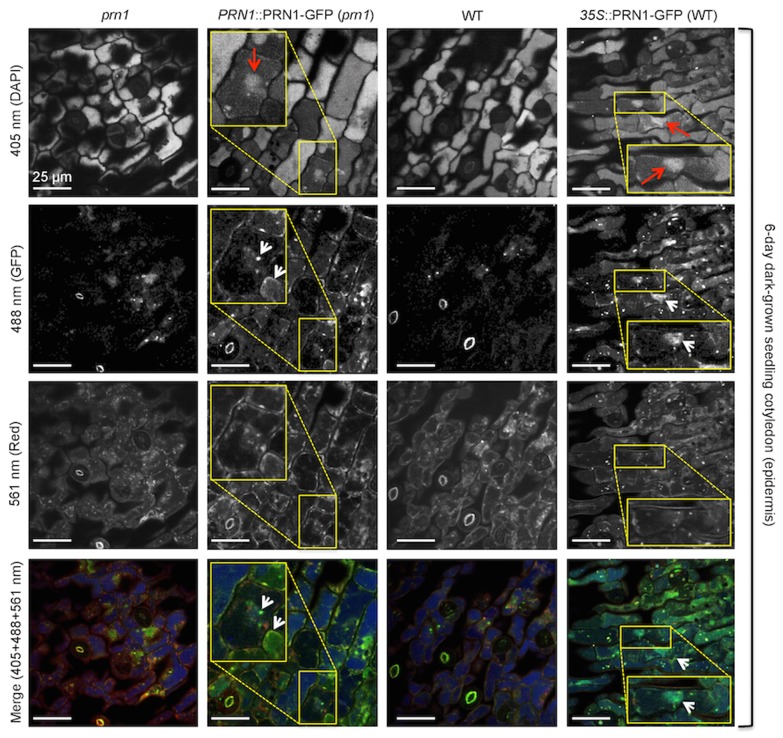
Subcellular localization of PRN1 in dark-grown seedlings. Constructs and transgenic plants are described in methods. 6-d-old dark-grown *prn1* whole seedlings transformed with *PRN1*::PRN1-GFP (T3) or WT seedlings transformed with *35S*::PRN1-GFP were fixed, stained with DAPI, mounted on slides, then photographed on a spinning disk confocal using steady state lasers 405 nm, 488 nm, and 561 nm. All images shown are from the cotyledon epidermis layer. Panel rows are indicated by wavelength, with individual channels in black and white. In the merge, 405 nm (DAPI) is false-colored blue, 488 nm (GFP) is false-colored green, and 561 nm is false-colored red (Red). Images are representative with no alteration of the fluorescence within the image field, and images represent an optical section of 1 μm thickness. The column heading indicates the different seedling lineages. Yellow boxes on the figure indicate areas of interest and show an enlargement. Red arrows indicate DAPI stain in nucleus. White arrows indicate GFP accumulation in 488 nm. Untransformed WT and *prn1* seedlings are also shown on the figure. Scale bars each represent 25 μm. n = 4 biological replicates, with at least 12–20 individual seedlings viewed per replicate; images are representative.

**Figure 5:**
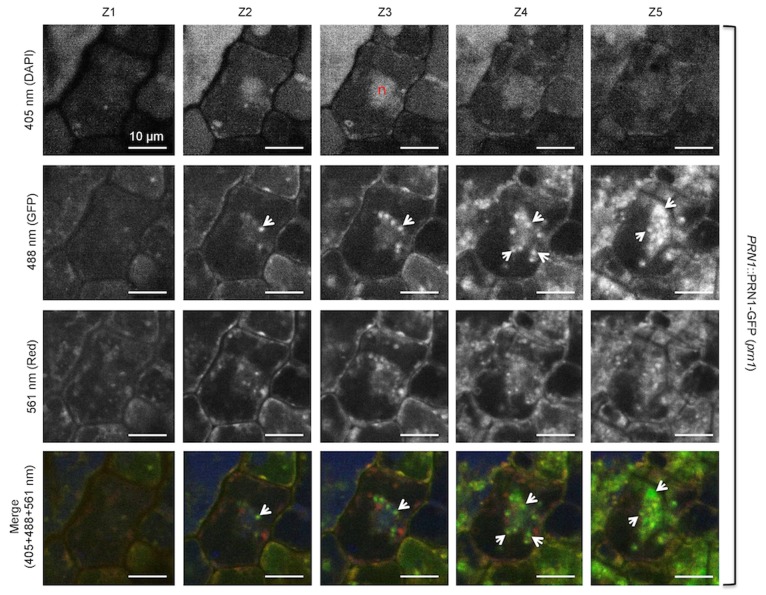
Enlargement and z-slices of region of interest in seedling cotyledon epidermis: *prn1* transformed with *PRN1*::PRN1-GFP. Microscopy and techniques are the same as described for Figure 4. Each row shows 5 consecutive z-slices. The red “n” indicates a nucleus (DAPI stained). GFP expression of interest is indicated by white arrows. Scale bar represents 10 μm.

**Figure 6:**
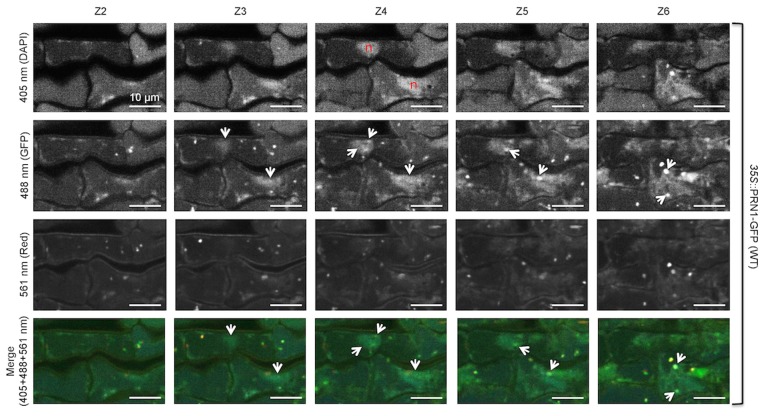
Enlargement and z-slices of region of interest in seedling cotyledon epidermis: WT transformed with *35S*::PRN1-GFP. Microscopy and techniques are the same as described for Figure 4. Each row shows 5 consecutive z-slices. The red “n” indicates a nucleus (DAPI stained). GFP expression of interest is indicated by white arrows. There is expression in the rest of the cell, largely diffuse. Scale bar represents 10 μm.

Subcellular localization of PRN1 was explored by utilizing PRN1-GFP flourescence. *prn1* mutant seedlings were transformed with *PRN1*::PRN1-GFP (T3), or WT seedlings were transformed with *35S*::PRN1-GFP (T2). Homozygous seedlings were grown under selection in complete darkness for 6 d, then images captured on a spinning disk confocal microscope. Flourescence was visible in some cells of the epidermis of the cotyledon, associated with nuclei (both diffuse and punctate), as well as diffuse within the cell cytoplasm of some cells in *prn1* transformed with *PRN1*::PRN1-GFP (Figure 4). Flourescence was greater in the transgenic plants overexpressing PRN1 (WT transformed with *35S*::PRN1-GFP) with localization to the nucleus, and diffuse and variable flourescence in the rest of the cell, visible in most of the cells of the epidermis (Figure 4). Areas of interest from Figure 4 images were enlarged to better observe the details over a series of 5 z-slices of 1 μm thickness (Figures 5 and 6). Enlargement of transformed (*PRN1*::PRN1-GFP) *prn1* (Figure 5) indicated fluorescence coinciding with DAPI stain throughout the nucleus, both diffuse and punctate. Flourescence was also observed immediately outside of the nucleus, possibly reflecting PRN1 trafficking to the nucleus or surrounding ER; diffuse and some punctate fluorescence was visible elsewhere in the cells shown (Figure 5). Some of the punctate fluorescence could correspond to developing plastids, as there are examples of fluorescence of GFP overlapping with 561 nm, which would be expected to cause etioplasts to fluoresce due to protochlorophyllides. Enlargement of the results for transformed WT (*35S*::PRN1-GFP) seedling (Figure 6) similarly revealed GFP fluorescence throughout the nucleus, with punctate structures within the nucleus, and fluorescence throughout the epidermal cells, diffuse and punctate. Like Figure 5, there is overlapping fluorescence with 561 nm in some examples. We also performed epidermal peel samples from living, non-fixed cotyledons, from seedlings grown 6 d in darkness or grown under 16∶8 (Figures S4 & S5). GFP-flourescence was largely found in the nuclei, and was diffuse in extranuclear locations.

### *PRN1* Promoter Possesses Regulatory Elements of Abiotic and Hormonal Signaling, and Some Specific Life Cycle Elements

The non-coding region upstream of the *PRN1* START codon (2,651 bp, inclusive of the 231 bp 3′ UTR end of At3g59210) to the gene upstream was cloned as the PRN1 native promoter, and was analyzed for regulatory elements and motifs (Figure S6; [38], [39]). The motifs indicate potential roles in carbon metabolism, hormone-signaling, light-signaling, and abiotic stress-responses, much of which is consistent with reported high-throughput data [24]–[26]. Lapik and Kaufman [21] also described aspects of the promoter 10 years ago, showing that there were indications for light and hormone regulation, specifically ABA, confirmed by exogenous-ABA regulation of the transcript and complementation [21]. The eFP browser [40] indicates that PRN1 is predicted to localize to the nucleus based on published experimental data and the WOLFPSORT prediction method, and possibly to the chloroplasts and cytosol, with a low-to-medium confidence value. In addition, PRN1 expression is shown to increase in response to ABA (guard cells), ACC, and abiotic signals, although conditions of seedling growth (age, sucrose in medium etc.) varied widely in the database [40].

## Discussion

Integration of a number of external environmental signals occurring simultaneously is necessary for seedling establishment. This transitional period from seed to seedling is complex, as limited materials including quercetins, potent antioxidants and other metabolites are stored in seeds to support seedlings until they become photosynthetically competent [41]. G-proteins and their effectors may be critical mechanisms to regulate this transition. Most higher plants only code for 1–2 copies of a G-protein-α subunit in the genome, yet there are numerous activities known to be associated with G-protein signaling in plants [27], [42]. This conundrum is often explained by pointing to the relatively large number of proposed effectors identified. The data presented herein indicate distinct and separable activities for a single effector, which may represent yet another dimension by which plant G-Protein signaling functions.

### GPA1 is a Possible Molecular Switch Regulating PRN1 Activity in the Cell

GPA1 is involved in many early processes in the seed-to-seedling transition ([43], [44], and reviewed in [27]). The data presented herein, coupled with previously published results [21], [22] indicate that the single-copy GPA1 may modulate PRN1 activity in Arabidopsis in multiple ways during seedling development. Given that GPA1 may spend a majority of time in the GTP-bound state [42], [45] we would speculate that PRN1 spends a reduced time or has less opportunity to act as a quercetinase (GPA1-GDP/inactive form). PRN1 may function primarily in the nucleus or perhaps in association with the nucleus (potentially the ER) shown herein, and at least in Arabidopsis, act as a transcription co-factor [22]. The quercetinase activity in the GPA1-PRN1 *in vitro* experiment appeared less than the PRN1-alone experiment (Compare Fig. 1B to Fig. 1A). Due to the importance of quercetin as an antioxidant and UV-screening molecule [3], [9], 46, and due to quercetin’s reported function as a transcriptional repressor [47]–[49], we would speculate that PRN1 may have reduced quercetinase activity in an organelle or cell, unless quercetin levels were attaining high or toxic levels. There are other proteins, as yet unknown, that may also interact with PRN1 or GPA1, altering PRN1 function(s). High-throughput yeast-2-hybrid assay data indicate that PRN1 interacts with a number of proteins [37]. However, separation of actual activities i.e. that of transcriptional regulator [22] versus quercetinase may be difficult to achieve, as Adams et al. have suggested in a review that pirins may have a role in protecting transcriptional machinery from the inhibitory effects of quercetin [50]. When considered with data of other organisms that show quercetin can affect transcription [47]–[49], [51], [52], it is possible that the transcriptional co-factor and quercetinase actions of PRN1 may be linked. Further studies are in progress by our lab group to better understand the functional relationships of PRN1 activities in plant cells.

### Regulation of Cellular Quercetin Levels and UV-induced Screening

PRN1 clearly affects quercetin levels in aerial portions of Arabidopsis (Figure 2). The changes that occur in the *prn1* mutant, increased quercetin and perhaps other flavonols or quercetin-derived structures, proved to be important in screening from high energy UV (Figure 2B,2C,2D). Several functions had been proposed for quercetin, but it is still not fully understood what chemical, or structural roles quercetin may play in the young seedling. Flavonoids play many diverse roles in plant responses to the external environment, and in protecting young seedlings from stress [46]. Quercetin, specifically, is known to be a potent antioxidant [3], a UV-screening compound [9], may be a regulator of auxin accumulation or transport [5]–[8], and even a modulator of transcriptional activity [47], [48].

### *prn1* Mutant and Transgenic Whole Seedling Responses to White Light

An additional phenotype of *prn1* mutants was revealed in this study. When *prn1* seedlings were grown under white light, hypocotyls deviated from WT gravitropic orientation (Fig. 3), a phenotype not observed under growth in continuous darkness. This disorientation was not a curvature, rather, the hypocotyls were straight, whether grown in 3-d continuous white light or 6-d under the 18∶6 growth protocol. Under the 16∶8 protocol at 6 d on vertical plates, it was also noticed that roots were shorter. WT seedlings transformed with *35S*::PRN1-GFP produced hypocotyls that were disoriented similar to *prn1* mutants, and seedling organs were all smaller than *prn1* mutants or WT–leaves, hypocotyls and roots. Both hypocotyl orientation and size of the seedling may be directly related to the changes that PRN1 may have on quercetin(s), where an alteration in quercetin levels may in turn affect auxin synthesis, metabolism or auxin transport.

Auxin influences cell division, cell elongation and cell size, and organ growth and development, and these auxin impacts are affected by flavonoids, reviewed in [53]. Poppe et al. described disorientation of the hypocotyl in far red light for WT Col, and a similar disorientation range for specific *phyB* mutants, in Landsberg and Nossen ecotypes [54]. The underlying cause of the disoriented hypocotyl phenotype of the *prn1* mutants is yet unknown, but this light-specific response may be due to a phytochrome effect on high quercetin levels in the *prn1* hypocotyl or the hypocotyl/root junction. Correct auxin transport is reported to be necessary for orientation and hypocotyl elongation in light-grown, but not dark-grown seedlings [55]. It was subsequently shown that there was a difference in auxin (IAA) transport in dark-grown versus light grown seedlings (lower in dark) [56]. In a detailed study of light affects on auxin transport and biosynthesis, Liu et al. showed that there is a difference in transport and metabolism of IAA between the upper (meristem, cotyledons and hook) and lower hypocotyl in tomato seedlings due to phytochrome action, and white light altered auxin transport of Arabidopsis seedlings, comparing basipetal to acropetal transport [57]. While we showed increased quercetin levels in *prn1* mutant shoots, in the overexpressor transformed seed lines (WT transformed with *35S*::PRN1-GFP), it would be expected that quercetins and perhaps flavonoids in general would be reduced from more PRN1 being present and potentially active as a quercetinase. It is envisioned that either way, increased or decreased, quercetin may affect directly or indirectly auxin transport. However, reduction or changes in ratios of flavonoid species (presumably in PRN1-overexpressor lines) on leaf size or development are difficult to predict without direct quantitation. How quercetin levels may affect scavenging of ROS due to IAA catabolism and resultant impacts on cell expansion would also be hard to predict [53], and hence the effects of overexpression of PRN1 will require additional experimentation to fully understand. Further studies are underway to quantitate PRN1’s regulation of quercetin levels in the shoot apex, cotyledon, hypocotyl, root-shoot junction and root itself. Subsequent effects on auxin synthesis, conjugation with molecular species of interest, and localization in the young seedling are also under investigation.

### Localization and Reconciliation of Activities of PRN1

PRN1 can act as a transcription co-factor [22] and quercetinase (herein), and has also been reported to interact with different proteins, including NF-Y [22] and GPA1 [21], [22], [37] where data indicate different subcellular locations. PRN1 was identified at multiple subcellular locations herein (Figures 4,5), where transcription cofactor activity would be expected to occur in the nucleus, but quercetinase activity could also occur. Quercetinase activity may be occurring at multiple intracellular sites, as Saslowsky et al. demonstrated that flavonoids and specific biosynthetic enzymes of flavonoids were present both in the nucleus and cytosol [10]. GPA1 is often discussed as a plasma-membrane localized protein, but it has also been found localized to the golgi and ER [58], indicating that there is opportunity for PRN1 to interact with GPA1 in multiple locations. The localization of PRN1 in the transformed *prn1* mutant indicates clusters of cells expressing PRN1-GFP. The fluorescence data possibly indicate trafficking of PRN1 in response to a local environmental signal, or perhaps a plasmodesmata-communicated signal, reminiscent of that observed for intercellularly-trafficked proteins in the leaf, for example, KNOTTED1 [59]. With a number of roles in the cell possible, the microenvironment may greatly affect expression or post-translational modification, linked to specific internal cues, or even associated with the cell cycle status, as has been reported in human cells [14], [60]. Quercetin is reported to be localized to many different parts of the cell [4], [10], perhaps to selectively carry out its many diverse functions.

### This G-protein-regulated Pathway may be Involved with Early Acclimation/Regulation of Cellular Stress Responses, both Abiotic and Biotic

GPA1 (and the Gα subunit of rice [61]) is reported to be involved in responses related to stress [62], [63] [64]. PRN1 interacts with GPA1 and as a result may play roles in responses to environmental stimuli. Quercetin is among the most abundant of the phenylpropanoids, and may assist in the prevention of damaging effects of many different types of abiotic and biotic stresses [46]. Enhanced levels of quercetin correlate with survival of *prn1* mutant seedlings after treatment with levels of UV-C normally lethal for WT, and the surviving seedlings, albeit shorter, do not appear to be damaged (Figure 2). This suggests that a major function of this signaling mechanism is to moderate protection of the young seedling from situations such as exposure to full sunlight, or the harmful radicals that can be generated via any cellular stress. While herein we have used UV radiation to study PRN1, it is possible that it may be activated by a host of stressors, biotic and abiotic. ABA is known to be a hormone involved in signaling related to different stress scenarios (reviewed in [68]), and *prn1* mutants have been reported to possess an enhanced sensitivity to ABA-inhibition of germination [21]. ABA involvement in the responses would be consistent with findings describing ABA activation of plant G-protein mediated pathways [42], [65]–[67] and our own data regarding B and ABA signaling [21], [22], [29]. Several recent studies have linked Pirins with the mechanisms of how plants are infected by various organisms, including Haustorium development in the in the parasitic plant *Triphysaria versicolor* [69] and survival of the human fungal pathogen *Cryptococcus neoformans* [23], the latter of which may be due to the LAC1 gene, where the *C. neoformans* laccase catalyzes the oxidation of quercetin [70]. Therefore PRN1’s role as a quercetinase, in a biotic context, may be influencing its biological functions. Consistent with a role in stress-mediation, quercetin was observed to bind the ER stress-induced kinase-endonuclease IRE1, and to function alongside stress signals from the ER lumen in modulating IRE1 activity in a yeast model [71]. The roles of PRN1 as a quercetinase and transcriptional co-factor, and its multiple sites of localization, may represent a plant-specific adaptation of multi-function, subcellular multitasking in higher plants.

## Supporting Information

Figure S1 **Analysis of the hypocotyl orientation phenotype of 3-d-old white light grown seedlings.** Seedlings were grown for 3 d in white light, and hypocotyl angle was measured for individual seedlings in reference to the horizontal phytatray surface (where vertical = 90°), n = 65. A non-parametric statistical analysis was performed, where the Kolmogorov-Smirnov normality test indicated that the WT and *prn1* data were not normally distributed (**1A.** WT: KS = 0.222, p<0.010, *prn1* **1B**. KS = 0.146 and p<0.010. **1C**. When *prn1* mutants were transformed with *PRN1*::PRN1-GFP (*PRN1*::PRN1-GFP (*prn1*)) and grown in white light for 3 d, the seedlings exhibited a restored WT hypocotyl orientation. WT and *prn1* seedlings are also shown on the figure. Seedlings on the phytatray were imaged from above. Scale bar = 1 mm.
(TIFF)

Figure S2 **Hypocotyl orientation responses of seedlings in 6-d complete darkness.** Seeds of WT, *prn1*, transformed line *PRN1*::PRN1-GFP (*prn1*) and transformed line *35S*::PRN1-GFP (WT) were sown on vertical plates, grown for 6 d in complete darkness, then photographed to view full seedling. Representative images are shown. Scale bar = 2 mm.
(TIFF)

Figure S3 **Hypocotyl orientation responses of seedlings in 6-d white light (16∶8).** Seeds of WT, *prn1*, transformed line *PRN1*::PRN1-GFP (*prn1*) and transformed line *35S*::PRN1-GFP (WT) were sown on vertical plates, grown for 6 d in white light (16∶8) then photographed to view full seedling. Representative images are shown. Scale bar = 2 mm.
(TIFF)

Figure S4 **Epidermal cotyledon peel of 6-d dark-grown seedlings indicates mainly nuclear localization.** Epidermal peels of live cotyledons of 6-d dark-grown seedlings of *prn1* mutants or *prn1*-transformed with *PRN1*::PRN1-GFP were viewed on spinning disk confocal after DAPI-stain. 10–15 cotyledons were viewed per replicate of 3 independent replicates. Images are representative. n = nucleus; scale bar = 25 μm.
(TIFF)

Figure S5 **Epidermal cotyledon peel of 6-d 16∶8-grown seedlings indicates mainly nuclear localization.** Epidermal peels of live cotyledons of 16∶8 dark-grown seedlings of *prn1* mutants or *prn1*-transformed with *PRN1*::PRN1-GFP or WT-transformed with *35S*::PRN1-GFP were viewed on spinning disk confocal after DAPI-stain. 10–15 cotyledons were viewed per replicate of 3 independent replicates. Images are representative. n = nucleus; scale bar = 25 μm.
(TIFF)

Figure S6 **Possible cis-regulatory elements of** ***PRN1*** **.** Frequently-repeated (≥20) cis motifs in the *PRN1* (At3g59220) promoter region (+ & − strand; 2,651 bp), determined from the “Database of plant cis-acting regulatory DNA elements” (http://www.dna.affrc.go.jp/PLACE/) [38], [39]. Y = T/C; N = G/A/C/T; R = A/G; W = A/T; nd = not determined. Each motif is represented by a symbol, and the approximate location of each repeat is displayed along the positive (+) and negative (−) strands of the *PRN1* promoter (from 5′ to 3′ direction). The 5′-UTR region of PRN1 is represented with an asterisk “*” (in the black box), and the beginning of the *PRN1* open reading frame is designated by its start codon start codon “ATG” (in gray box). The white part of the 5′ to 3′ bar represents the 3′UTR (223 bp) of At3g59210, a gene that putatively codes for a protein with homology to F-box/RNI-like superfamily, cyclin-like, and LRR2 proteins.
(TIFF)

Table S1 **Primers used for cloning.**
(TIFF)
