# Supplementary figures and images for: Pirin1 (PRN1) Is a Multifunctional Protein that Regulates Quercetin, and Impacts Specific Light and UV Responses in the Seed-to-Seedling Transition of Arabidopsis thaliana
Source: PLoS One. 2014 Apr 4;9(4):e93371. doi: 10.1371/journal.pone.0093371 (PMC3976398; doi:10.1371/journal.pone.0093371)

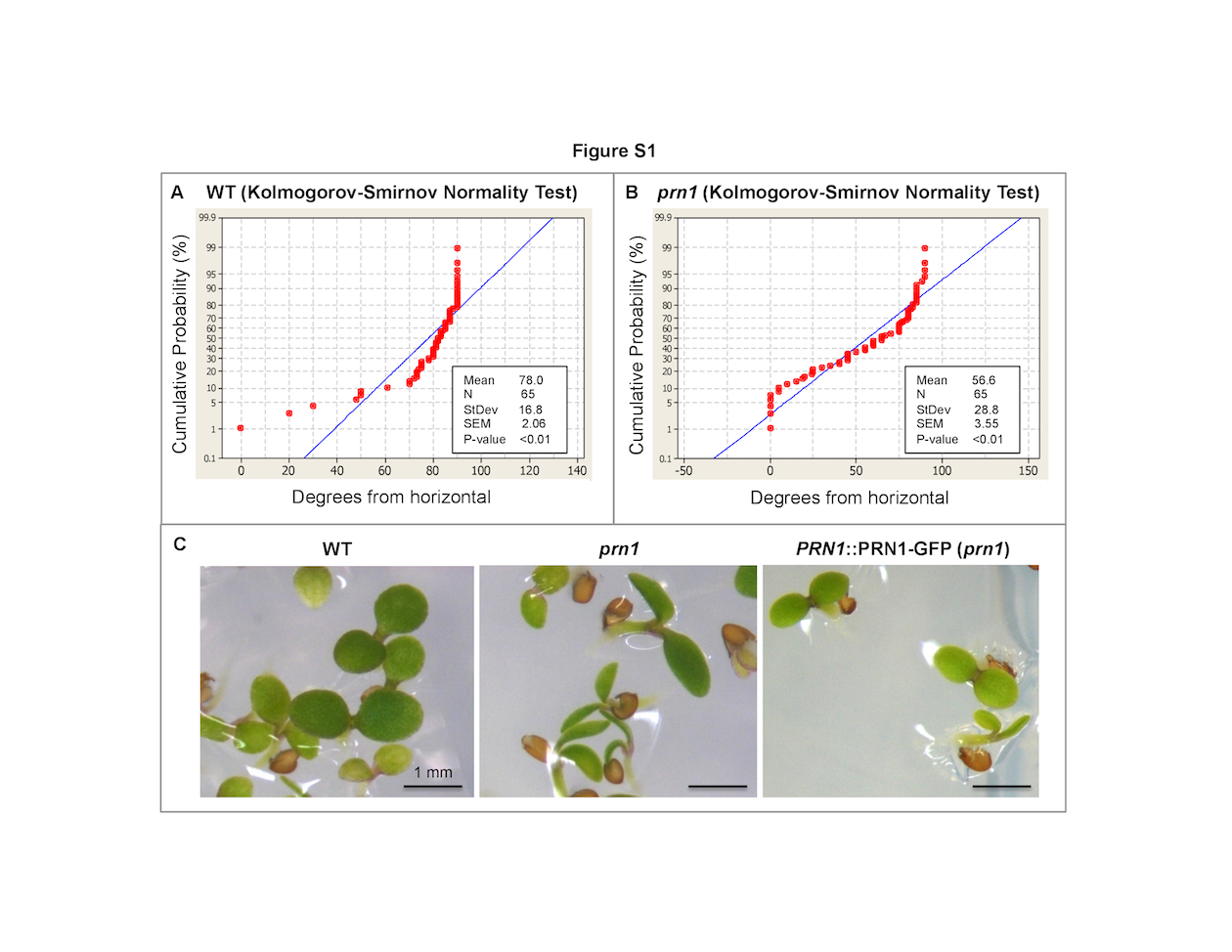

Supplement: Figure S1 — Analysis of the hypocotyl orientation phenotype of 3-d-old white light grown seedlings. Seedlings were grown for 3 d in white light, and hypocotyl angle was measured for individual seedlings in reference to the horizontal phytatray surface (where vertical = 90°), n = 65. A non-parametric statistical analysis was performed, where the Kolmogorov-Smirnov normality test indicated that the WT and prn1 data were not normally distributed (1A. WT: KS = 0.222, p<0.010, prn1 1B. KS = 0.146 and p<0.010. 1C. When prn1 mutants were transformed with PRN1::PRN1-GFP (PRN1::PRN1-GFP (prn1)) and grown in white light for 3 d, the seedlings exhibited a restored WT hypocotyl orientation. WT and prn1 seedlings are also shown on the figure. Seedlings on the phytatray were imaged from above. Scale bar = 1 mm. (TIFF) [file pone.0093371.s001.tif]

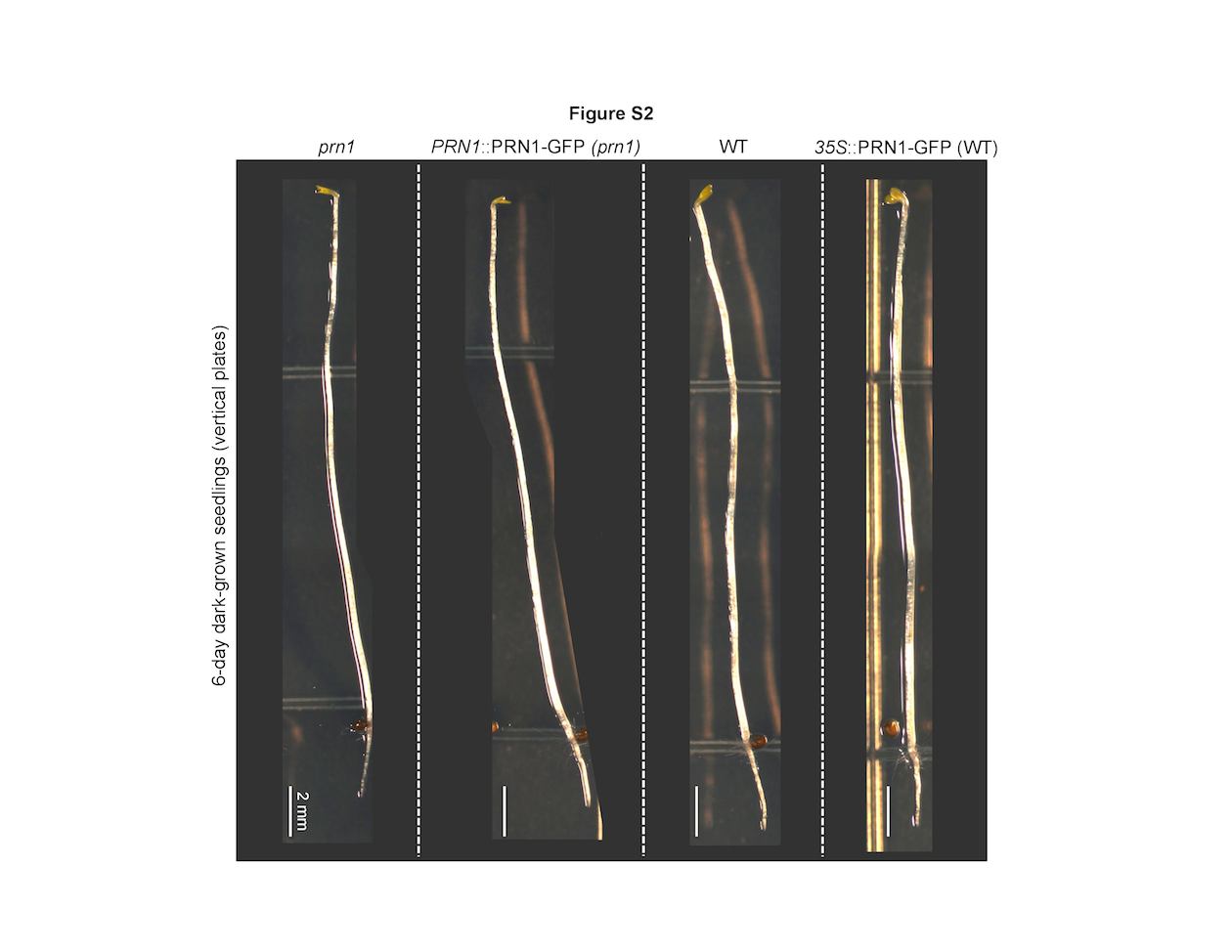

Supplement: Figure S2 — Hypocotyl orientation responses of seedlings in 6-d complete darkness. Seeds of WT, prn1, transformed line PRN1::PRN1-GFP (prn1) and transformed line 35S::PRN1-GFP (WT) were sown on vertical plates, grown for 6 d in complete darkness, then photographed to view full seedling. Representative images are shown. Scale bar = 2 mm. (TIFF) [file pone.0093371.s002.tif]

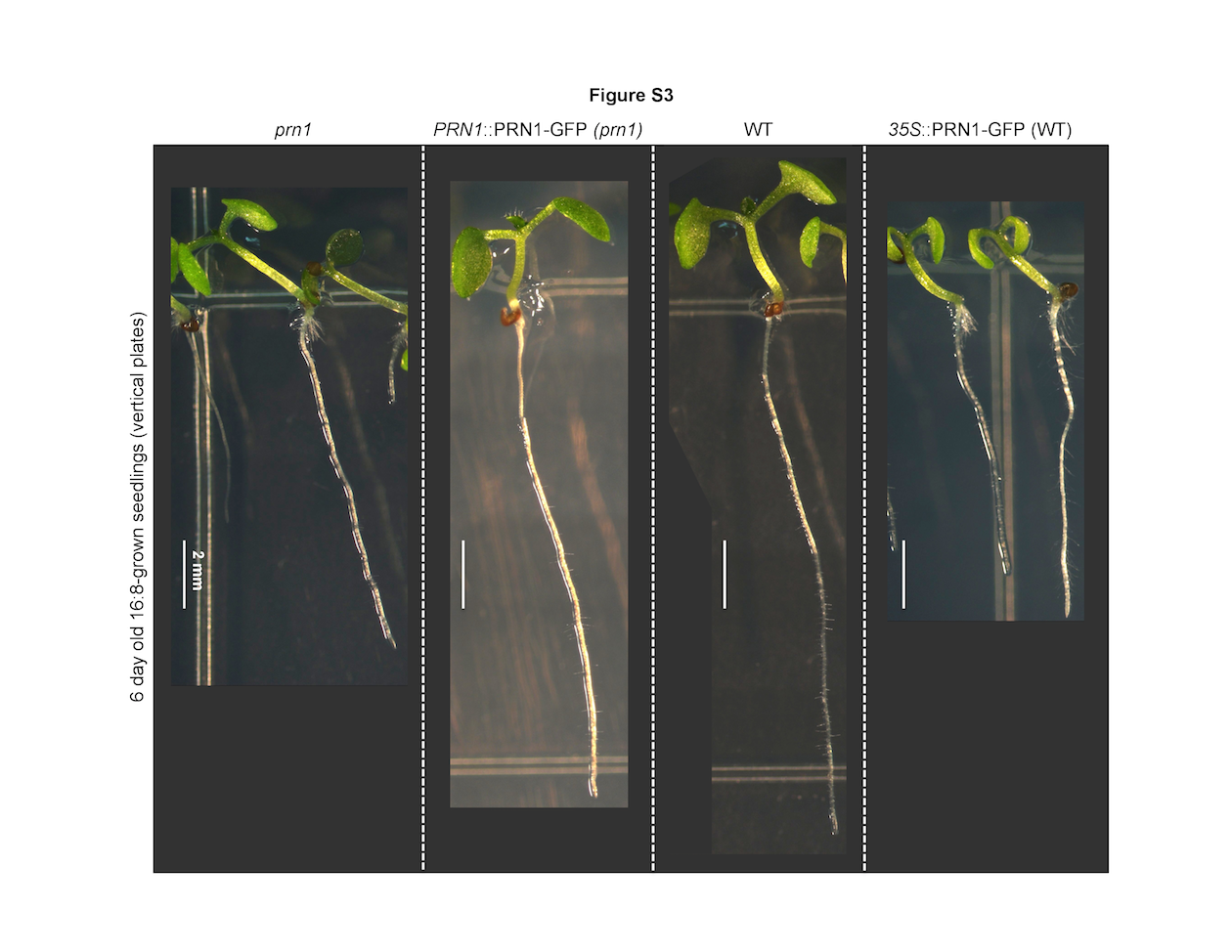

Supplement: Figure S3 — Hypocotyl orientation responses of seedlings in 6-d white light (16∶8). Seeds of WT, prn1, transformed line PRN1::PRN1-GFP (prn1) and transformed line 35S::PRN1-GFP (WT) were sown on vertical plates, grown for 6 d in white light (16∶8) then photographed to view full seedling. Representative images are shown. Scale bar = 2 mm. (TIFF) [file pone.0093371.s003.tif]

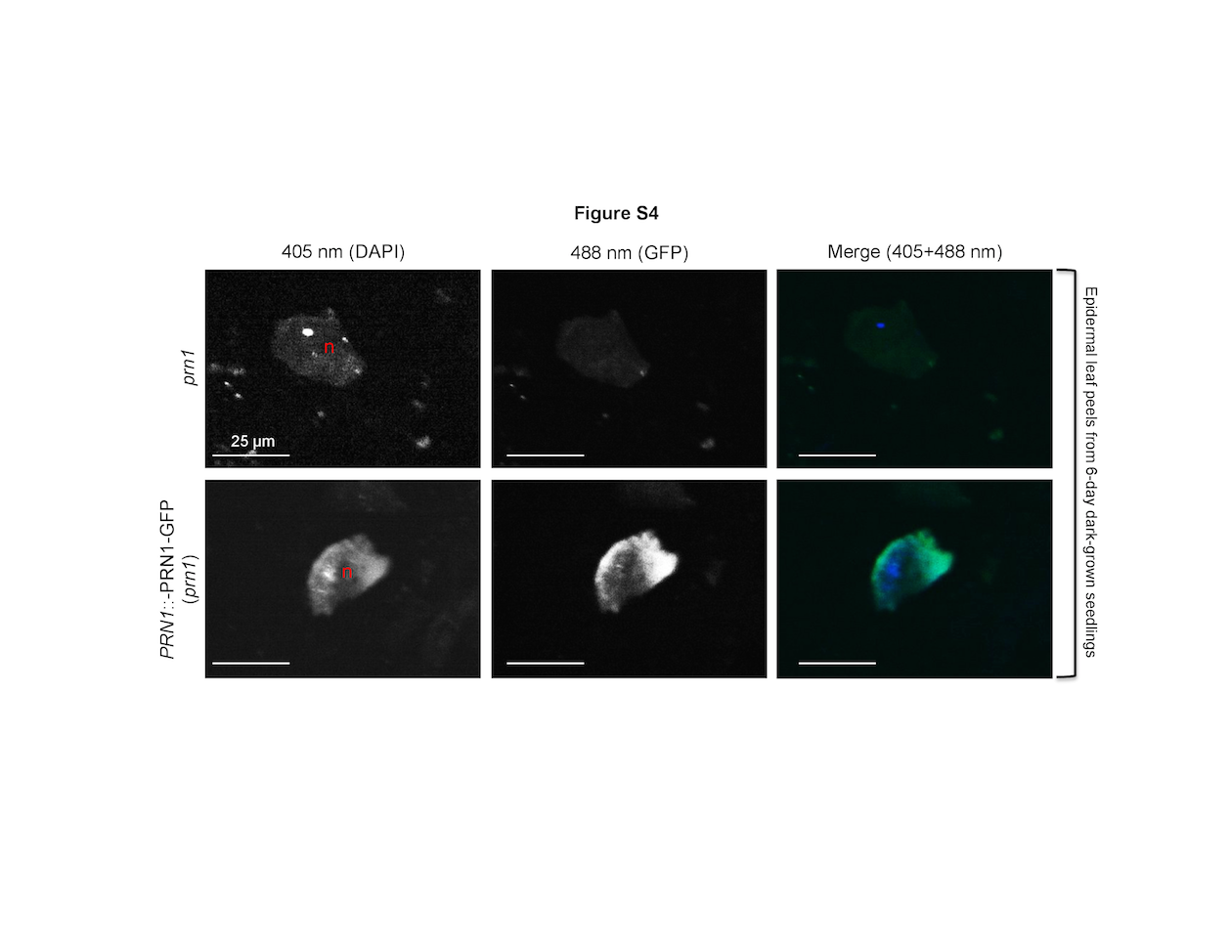

Supplement: Figure S4 — Epidermal cotyledon peel of 6-d dark-grown seedlings indicates mainly nuclear localization. Epidermal peels of live cotyledons of 6-d dark-grown seedlings of prn1 mutants or prn1-transformed with PRN1::PRN1-GFP were viewed on spinning disk confocal after DAPI-stain. 10–15 cotyledons were viewed per replicate of 3 independent replicates. Images are representative. n = nucleus; scale bar = 25 μm. (TIFF) [file pone.0093371.s004.tif]

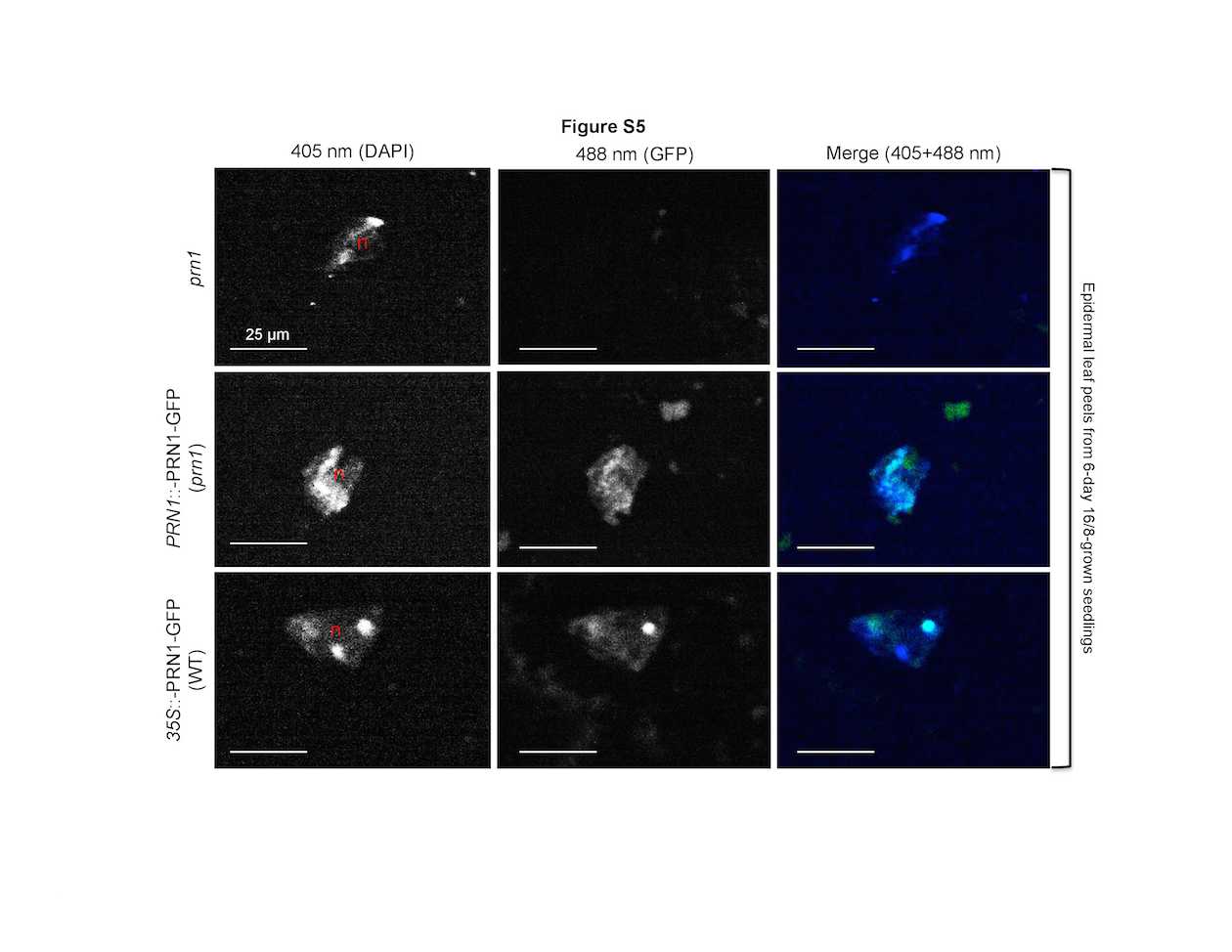

Supplement: Figure S5 — Epidermal cotyledon peel of 6-d 16∶8-grown seedlings indicates mainly nuclear localization. Epidermal peels of live cotyledons of 16∶8 dark-grown seedlings of prn1 mutants or prn1-transformed with PRN1::PRN1-GFP or WT-transformed with 35S::PRN1-GFP were viewed on spinning disk confocal after DAPI-stain. 10–15 cotyledons were viewed per replicate of 3 independent replicates. Images are representative. n = nucleus; scale bar = 25 μm. (TIFF) [file pone.0093371.s005.tif]

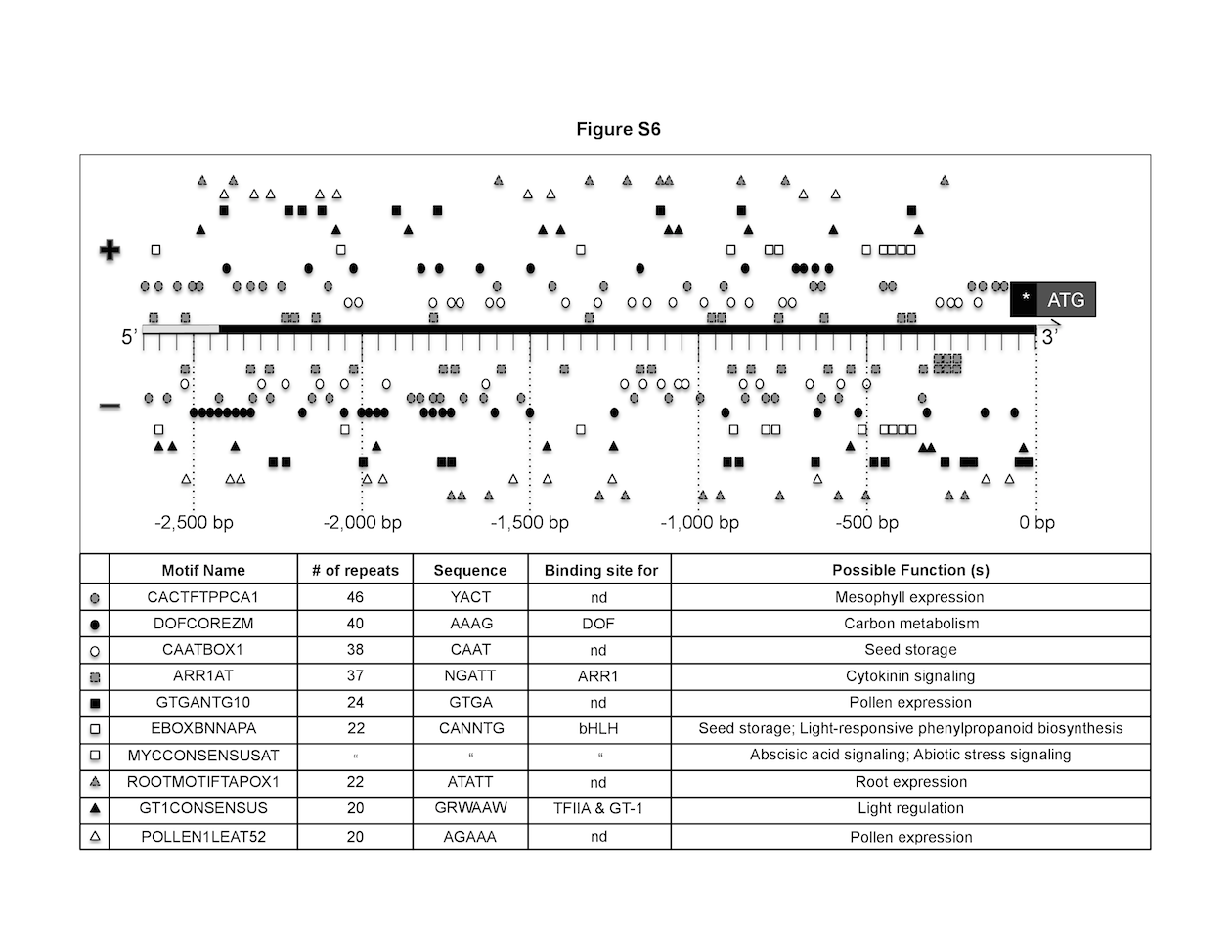

Supplement: Figure S6 — Possible cis-regulatory elements of PRN1 . Frequently-repeated (≥20) cis motifs in the PRN1 (At3g59220) promoter region (+ & − strand; 2,651 bp), determined from the “Database of plant cis-acting regulatory DNA elements” (http://www.dna.affrc.go.jp/PLACE/) [38], [39]. Y = T/C; N = G/A/C/T; R = A/G; W = A/T; nd = not determined. Each motif is represented by a symbol, and the approximate location of each repeat is displayed along the positive (+) and negative (−) strands of the PRN1 promoter (from 5′ to 3′ direction). The 5′-UTR region of PRN1 is represented with an asterisk “*” (in the black box), and the beginning of the PRN1 open reading frame is designated by its start codon start codon “ATG” (in gray box). The white part of the 5′ to 3′ bar represents the 3′UTR (223 bp) of At3g59210, a gene that putatively codes for a protein with homology to F-box/RNI-like superfamily, cyclin-like, and LRR2 proteins. (TIFF) [file pone.0093371.s006.tif]

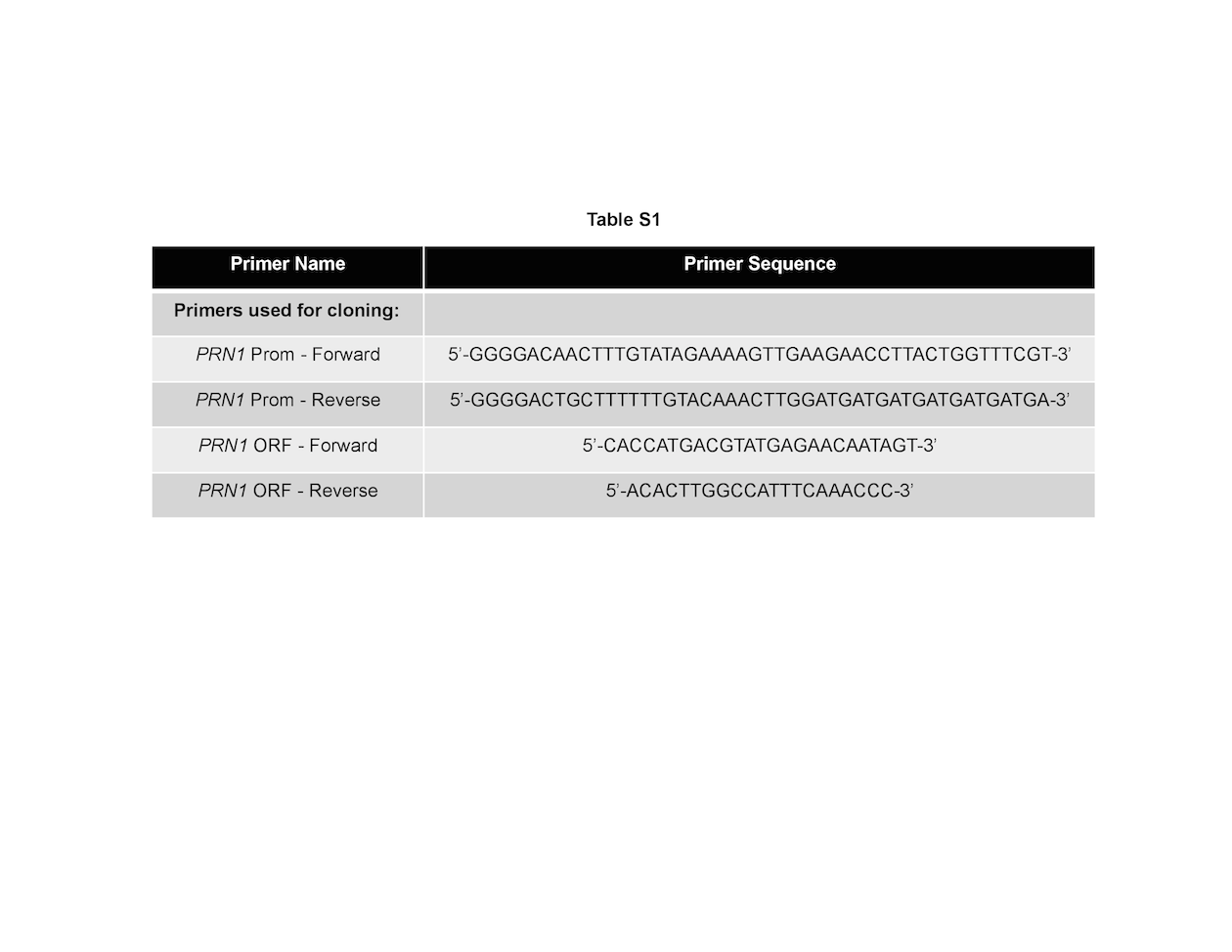

Supplement: Table S1 — Primers used for cloning. (TIFF) [file pone.0093371.s007.tif]
